# Implementing Reproducible Fisheries Research: A Decade of Experience With the Kahawai Reporting System

**DOI:** 10.1002/snz2.70011

**Published:** 2026-02-04

**Authors:** David A. J. Middleton, Finlay N. Thompson, Adam D. Langley, Philipp Neubauer

**Affiliations:** ^1^ Pisces Research Limited Wellington New Zealand; ^2^ Dragonfly Data Science Wellington New Zealand; ^3^ Trophia Limited Nelson New Zealand

**Keywords:** continuous integration, data analysis, fisheries, reproducibility, research integrity, transparency, trust, version control

## Abstract

Scientific publishing is widely perceived to be in a state of crisis. A contributing factor is reproducibility: the extent to which the results can be replicated is key to assessing the reliability of a study. Reproducibility rates have often been found to be low, a situation complicated by the fact that many studies provide insufficient details of their methods.

For the last decade, we have been using a framework that allows us to fully reproduce the results of analyses of fisheries data. Key features are that the analyses are fully defined in code and run within a consistent computing environment. The approach has proven to be transferable, with the framework being adopted in a range of other disciplines.

Incentivising the broader adoption of fully reproducible analyses would assist in re‐establishing trust in scientific publications, addressing the “reproducibility crisis” while also providing a basis for strengthened peer review processes and increasing the likelihood that questionable research practices are identified.

Implementing fully reproducible analyses has some overhead, especially where the software tools that support the approach are unfamiliar. We have found that the benefits of openness, transparency, and efficiency, together with increased collaboration, make this overhead worthwhile.

## 1 Introduction

Bolland et al. (2025) observe that “trustworthy literature is an essential part of knowledge, evidence‐based information, and science”. Yet there is growing evidence that much of the published scientific literature is *not* trustworthy. We sympathise with the researcher who stated “you can't just read an abstract and have any faith in it. I kind of assume everything's wrong” (Joelving et al. 2025).

Bolland et al. (2025) describe issues that compromise the integrity of scientific publications, ranging from trivial, unintentional mistakes to fabrication and fraud. In addition to human factors, the role of generative AI in producing manuscripts, e.g., Cabanac and Labbé (2021); Van Noorden (2023), and peer reviews (Zou 2024) is a growing issue.

Failings of the peer‐review system and the slow propagation of corrections have been highlighted in the fisheries literature for at least two decades (Hilborn 2006; Cochrane et al. 2024). Similar sentiments are expressed in other fields of research; for example, Bolland et al. (2025) focussed on biomedicine. Trueblood et al. (2025) suggest that many of the problems in contemporary scientific publishing can be attributed to the incentives inherent in the “academic prestige economy” and discuss alternative publication and academic evaluation models that are intended to be better aligned with the reliable dissemination of knowledge.

In New Zealand, most operational fisheries research conducted to monitor stocks and inform management decisions, including setting catch limits, is funded (directly, or indirectly via cost recovery) by those who own the rights to harvest catch. This funding model is generally considered to have created appropriate incentives, but has also created a range of challenges (Stokes et al. 2006; Harte 2007; Mace et al. 2014; Baker et al. 2023). Despite Fisheries New Zealand's science standard (Ministry for Primary Industries 2011) that puts peer review as the central mechanism for establishing science quality, building on long‐established science working group and plenary review processes (Maunder and Starr 2002; Hurst 2014), the involvement of seafood industry funded scientists is often assumed to create bias (see, for example, Slooten et al. 2017, noting the response by Melnychuk et al. 2017).

A key part of establishing whether research is reliable is confirming that it is repeatable. Reproducibility rates as low as 10% have been reported from some subject areas, and a 2016 survey suggested that more than 70% of researchers had tried and failed to reproduce another scientist's experiments (Baker 2016). One commentator in that survey indicated that “at the current time there is no consensus on what reproducibility is or should be”. The science standard for New Zealand fisheries (Ministry for Primary Industries 2011) indicates that it should be possible to “*demonstrate* that results may be reliably reproduced by an independent scientific expert using the same data and analytical methods” (our emphasis), but gives no specific guidance on how reproducibility can be routinely demonstrated.

Gundersen (2021) agreed that reproducibility was an elusive concept and provided the following definition:

Reproducibility is the ability of independent investigators to draw the same conclusions from an experiment by following the documentation shared by the original investigators.

Gundersen (2021) further suggested that there are three “degrees of reproducibility” (termed outcome, analysis and interpretation reproducibility), and concluded that the level of reproducibility that is achievable is dependent on the completeness of the documentation of the original experiment and analysis. This led to the definition of four “reproducibility types” depending on whether the documentation comprises a textual description of the experiment, analysis code, the data produced, or all three. Gundersen (2021) emphasised that transparency and openness were key contributors to reproducibility.

In many fields, including fisheries stock assessment but also population ecology more generally, observational research is more common than experimental approaches. In this situation, once data have been collected, then the results of any given analysis should be perfectly repeatable, so long as the documentation of that analysis is adequate. Our experience is that this is often not the case, principally because “written language is a poor substitute for code” (Gundersen 2021).

Repeating the same analysis with the same code does not completely meet the definition of reproducibility outlined by Gundersen (2021); for example, it is also appropriate to ask whether different coding of the analysis, or different statistical methods, would lead to the same conclusion. Nonetheless, achieving repeatability is often handicapped by authors providing insufficient detail on their methods (or the code used).

Different research fields have developed different standards for documenting analyses; we have found the genetics literature particularly challenging, as it is common to “document” the analytical methods by simply citing the software used, without further detail on the algorithm or even the software settings. Simoneau et al. (2021) demonstrated that this issue was pervasive in studies using ribonucleic acid sequencing, where the associated bioinformatics pipeline was usually insufficiently specified; they reported that only 25% of studies described all the required computational steps, and even fewer reported the parameter values required to ensure complete reproducibility.

Fidler and Wilcox (2021) suggest that the "reproducibility crisis", while challenging, has inspired work to strengthen the foundations of science. Here, we describe the framework that allows us to undertake *fully repeatable* analyses of fisheries data. It uses a range of modern computing tools but, fundamentally, relies on the discipline of ensuring that an analysis is completely defined in code. We consider this meets the Fisheries New Zealand requirement for demonstrably reproducible analyses, but also has wider applicability in addressing a lack of trust in the scientific literature.

## 2 An Infrastructure for Repeatable Analyses

Here we discuss the principles, and an associated infrastructure (the kahawai.io platform), that allow us to have confidence that our research outputs are fully reproducible. Fundamentally, the process is simple: a reproducible analysis must be completely defined in code, such that running a single command produces the same outputs when run, unattended, multiple times. We return to the question of assessing whether outputs are the same below, and first focus on the requirement that the analysis is fully coded.

### 2.1 Fully Encoded Analyses

An analysis fully defined in computer code, so it runs without any interaction after launching, ensures that the results are not dependent on human interaction (for example, setting a software parameter), and are therefore insulated from the inevitable errors that such interactions produce. Some errors are simple mistakes, such as occasionally setting a parameter incorrectly, but others may arise because the analysis is still being developed: for example, “tuning” a parameter to improve how an algorithm operates, but forgetting to record the final parameter value used.

Quantitative analysis involves making many small decisions regarding data, each of which is associated with some form of justification or discussion referring back to the objectives of the analysis. It is important to record these decisions as they are made. If the analysis is implemented in code, it is easy to document the decisions directly, either through the code itself or with accompanying comments.

From the perspective of the analyst, a reproducible, fully encoded analysis operates in “batch mode” (the computer works unattended) rather than “interactive mode” where the analyst guides the analysis via a user interface.

The requirement for analyses that are fully specified in code inevitably affects the type of software that is typically used in reproducible analyses. For example, specifying a statistical analysis via code using the R language (R Core Team 2024) is generally preferred over the use of a spreadsheet, where an analysis would usually require selection from a menu and entering of parameters in a subsequent dialogue box.^1^

### 2.2 Version Control for Code

A requirement that analyses be fully specified in code leads to the need for a version (or revision) control system. It is extremely rare that the code for an analysis is completely correct the first time, even in the most trivial of cases. Exploratory data analysis, including visualising patterns in the data, should be a key part of any analysis. While it is simple to write code to read in a data file and, say, compute the mean value, we would advocate for inspecting the distribution of those data first, if for no other reason than ensuring that data entry errors (such as missed decimal points) are not present.

The need for exploratory data analysis, and the fact that most analyses will be complex, involving multiple steps, implies that an analysis will be run many times before it is finalised (see our discussion of a continuous integration approach, below). Sometimes the development of an analysis will proceed in a manner that is subsequently found to be incorrect, requiring stepping back to an earlier point. All of these considerations imply that use of a version control system is a necessary prerequisite for reproducible analyses. The need for version control of analysis code has been noted for some years (see, for example, Gentleman and Temple Lang (2007). Hamra et al. (2025) highlight the range of version control software currently available, and the benefits of adopting these early in the development of a reproducible analysis rather than simply using them as an archiving tool once the code for an analysis has been finalised.

### 2.3 Reproducible Computing Environments

Having analyses fully specified in computer code is a necessary, but not sufficient, part of achieving reproducible analyses. This is because the results of running a piece of code can vary in different computing environments. In some cases, it may simply fail to run at all: for example, if the required software is not available, or if different versions of software behave in different ways. In other situations, the results may depend on the computer architecture (i.e., the hardware) on which the code is run (Goldberg 1991), although the adoption of standard numerical libraries, which have been tested on multiple architectures, means that hardware differences are less likely to lead to differences in analyses using modern software designed to run on multiple platforms.

Completely specifying the computing requirements to reproduce an analysis can be challenging. Many analyses require a suite of additional libraries or packages, in addition to the main software used. Attempting to provide a comprehensive list of software versions used is prone to errors of omission, and generates lists that would fall foul of the word count limits inherent in scientific publication and are not actually useful to those attempting to reproduce an analysis because they lack a mechanism for implementation. Instead, what is required is a way to ensure that an analysis can be rerun in the same computing environment used to produce the original results.

Our solution to ensuring that code can be rerun in an identical computing environment has been the use of Docker images (Merkel 2014). Boettiger and Eddelbuettel (2017) provide a straightforward introduction to Docker from the perspective of running the R statistical software; here, we simply note that running the code for an analysis using a Docker image ensures (subject to some caveats we discuss later) that the code is run in the same computing environment as the original analysis. The Docker image effectively packages the operating system, software, and libraries into a single binary package that can be run on any Docker host.

In principle, Docker images can be produced in a reproducible manner by carefully specifying the versions of all software that is added to the image. In practice, it is harder to guarantee reproducible image builds because of the difficulty in guaranteeing that the required versions of software will be available in future from internet sources that are managed by others.

Our practice has been to specify the “recipe” for building a Docker image, and to maintain an archive of Docker images that result from running the recipe at different times. This serves two purposes: the exact image that was used in an analysis is available on a long‐term basis, and the occasional updating of the image used can contribute to establishing reproducibility in the broader sense envisaged by Gundersen (2021), with slight variation in the analyses and conditions.

### 2.4 A “Continuous Integration” Approach to Analysis

We suggest that running a fixed set of code, on a static set of data, and in a fixed computing environment, such as a Docker image, should be considered sufficient to ensure that an analysis is reproducible (although, see caveats in the Discussion below).

In documenting how to implement reproducible analyses, we have relied on tools, such as version control systems, that were originally developed for software engineering and have been used by software engineers for decades. Continuous delivery systems are now standard practice in the software industry, although not without challenges as well as benefits (Pinto et al. 2018). The central idea of continuous delivery is that software systems should be continuously built and automatically tested to ensure that any errors introduced in new code are identified and fixed as soon as possible. In the context of reproducible scientific analyses, this means that every time the code is changed, the analysis should be rerun *from the beginning*.

Without a continuous integration approach, data analyses will often proceed incrementally, with development focusing on data objects produced by earlier analyses. In this approach, it is not uncommon for some steps to be forgotten and omitted from the analysis code. Implementation of a continuous integration approach, where the analysis is periodically rerun from the start, relying only on the raw data inputs, helps to ensure that the analysis remains fully reproducible.

## 3 The kahawai.io System

**FIGURE 1:**
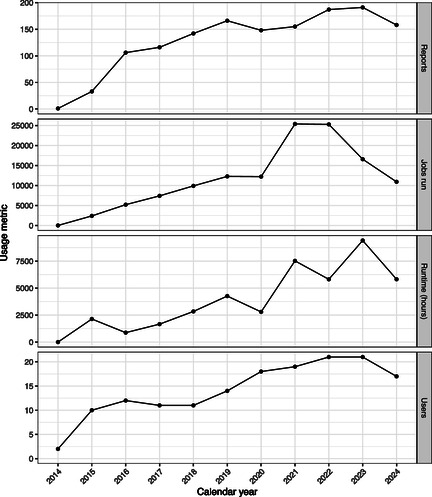
Usage of the kahawai.io system from 2014 to 2024. **Reports** is the number of reports run each year, where a “report” is a collection of version‐controlled code that produces a particular output; **Jobs run** is the number of individual builds of these reports, irrespective of whether the job completed successfully, with **Runtime (hours)** the amount of compute time involved. **Users** is the number of people running reports on the system.

The Kahawai reporting system (kahawai.io) provides a continuous integration and delivery approach to analyse and report from New Zealand fisheries databases. Initially implemented in 2014, it has been in continuous use since then, with usage increasing over time (Figure 1).

### 3.1 System Components

**FIGURE 2:**
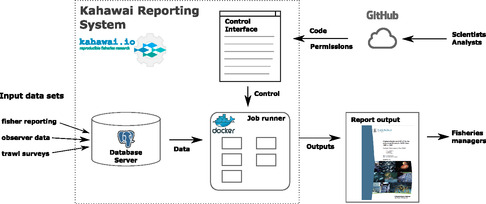
Components of the Kahawai reporting system. The dotted border contains the reporting system core infrastructure, while code, input data, and outputs are indicated by arrows crossing that border.

The system includes three components: a database server, a job runner, and a control interface (see Figure 2). Scientists interact with the system through code committed to the GitHub^2^ version control system, and through the control interface. Outputs are available through the control interface. These components are described in more detail in the Supporting Information.

### 3.2 Advantages of the Kahawai Reporting System

Although we have introduced the kahawai.io system in the context of reproducible research, it supports a number of other important high level aims that we characterise as *efficiency*, *openness*, and *confidence*.

Efficiency is gained by streamlining the generation of reports, delivering more frequent and faster reporting on fisheries. The kahawai.io system also aims to encourage sharing and collaboration, building a culture of openness both within its user community (see the discussion of the Kahawai Collective in the Supporting Information) and across the wider fisheries science community. Finally, by ensuring that all activity can be audited and checked, the system ensures that the science community, fisheries managers, and the wider public can have confidence in the reporting.

### 3.3 Efficiency

The kahawai.io system has provided efficiencies in two key areas: one specific to the New Zealand fisheries application, and one more generic. In the fisheries context, the fact that the kahawai.io system provides access to a reasonably complete version of Fisheries New Zealand's databases, specifically set up for the analyses we routinely undertake, is the key efficiency. Fine‐scale fisheries data are confidential, and Fisheries New Zealand must give specific permission for their use in any given project. However, given receipt of that permission, the data are available immediately via the kahawai.io system, in a consistent format.

Building a research version of the Fisheries New Zealand databases also provides the opportunity to apply routine data preparation approaches. Following the establishment of the Quota Management System in 1986, New Zealand has amassed a rich dataset of comprehensive information from commercial fishing activity. However, data collection has evolved: from aggregated data to finer scales, and from the use of paper forms to electronic reporting. To use these data as a consistent time series, one has to account for the changes in data collection and address the inevitable errors that occur. The application of standardised procedures, developed after use of these data in a wide range of projects, to the database at build time saves having to apply these to each analysis individually.

There are potential downsides to providing analysts with access to datasets that have been prepared in a standardised way. In particular, while it provides new analysts with a head start in undertaking useful projects, it risks not exposing them to the data in their rawest form, and so gaining an understanding of why and how the data preparation rules are applied. We consider that this risk is mitigated in two ways: ensuring that the data preparation code is available for inspection and critique, and specifically encouraging all users of the system to be engaged in continuous improvement of the data preparation approaches.

The more generic efficiency from the use of a system like kahawai.io accrues from the ability to share code and collaborate on problems. For example, taking advantage of the consistent data formats available from the kahawai.io database, we have co‐developed code libraries that allow us to efficiently undertake a range of standard fisheries analyses. Rather than use these as static resources, we find that these are continually improved over time; for example, to address new data types, apply new approaches generically rather than specifically, take advantage of improved downstream libraries, or simply produce better graphical output to aid in data presentation.

In terms of personal scientific endeavour, the kahawai.io system has provided us with the time to pursue more innovative analyses while still delivering the key outputs that inform fisheries managers.

### 3.4 Openness and Collaboration

As noted by Gundersen (2021), openness and collaboration are fundamental to reproducibility in science. Frequently, openness is provided solely through the publication of the methods and results of an analysis in the scientific literature, and collaboration may be quite limited—even with those who specifically attempt to reproduce the work (Baker 2016).

Implementing a fully reproducible analysis, by ensuring that all the steps required are documented in code, supports collaboration (even if only with one's past self when aiming to repeat or extend a previous analysis). However, our experience has been that coding fully reproducible analyses, especially where these make use of the efficiencies described above, encourages and supports collaboration with colleagues. This is assisted by adopting consistent approaches to delivering projects (see “Conveniences that build consistency” in the Supporting Information).

### 3.5 Confidence and Transparency

Fisheries managers, both from government agencies and seafood industry bodies, are the primary consumers of fisheries research in New Zealand. Although direct access by fisheries managers to the Kahawai reporting system is relatively rare, it is not unusual for us to show the system in use as we respond to specific information requests. In some cases, those managers may wish to review and even interact with the reporting process. The system makes it easy for small changes to the text, or the reparametrisation of an output, to be made independently of the scientists. Because any changes can be reviewed in the code repository log, there is less risk that errors are introduced or inappropriate changes made.

More importantly, in a climate where the results of fisheries analyses can be controversial and lead to debates (sometimes politicised) between stakeholders affected by decisions made on the basis of these analyses, the approach we have implemented provides us with the assurance that any analyses we undertake can be independently scrutinised.

### 3.6 Ongoing Development

Because kahawai.io has become an integral part of the work undertaken by several fisheries research providers, we have established the nonprofit Kahawai Collective to maintain and develop this system. The control interface used by the kahawai.io system (Gateaux) has also been adopted at a number of New Zealand government agencies including the Ministry for Business, Innovation, and Employment (MBIE), the Department of Conservation (DOC), and the Ministry for Primary Industries (MPI). It has also been adopted by the South Pacific Regional Fisheries Management Organisation (SPRFMO), and the Western Central Pacific Fisheries Commission (WCPFC). The Supporting Information provides further information on both these aspects.

## 4 Discussion

The Kahawai reporting system (kahawai.io) has been in active use for the last decade. It is well tested and, as demonstrated by other groups adopting the Gateaux software, it is a reproducible model for reproducible research. Although we have refined some aspects of the system, the basic infrastructure has been in place for the duration. Outputs from every job run on the system are stored and can still be accessed if required. We now use the kahawai.io system (or other Gateaux‐based systems) for virtually all our work. There is, however, variation in the extent to which any given project implements the fully reproducible approach we describe. In some cases, the full project—including the final report—is fully reproducible in the kahawai.io system, but in other cases, it is only used for part of a project.

The fact that our analyses are implemented to a demonstrably high standard of reproducibility is reassuring, and a discipline that we consider should be more widely adopted, but the practical benefits in terms of increased efficiency and collaboration are probably the more tangible reasons that we would encourage other researchers to face the—admittedly potentially steep—learning curve of adopting the approaches described here. Our experience matches that of Alston and Rick (2021) who suggest that reproducible research primarily benefits those who do it, although clearly the wider research community also benefits.

Similar approaches to reproducible analyses have been independently implemented by a number of other groups worldwide; for example Beaulieu‐Jones and Greene (2017) describe a Docker‐based, continuous integration approach to routine analyses of differential gene expression, while Essawy et al. (2020) describe a more bespoke approach focussed specifically on encapsulating data analyses and modelling (but development of the latter approach appears to have ceased). However, these approaches are not broadly known or adopted (i.e., they are not normal scientific practice), largely because the benefits are not always clear to those in the “academic prestige economy” that rewards publication speed and volume rather than demonstrated adherence to a reproducibility standard. With the need to restore confidence in the scientific literature, we consider that requiring demonstrably reproducible analyses will be a worthwhile and necessary exercise.

Raising the standard of scientific practice is not necessarily straightforward. An editorial in *Nature* (Adam 2023) drew attention to an experimental study that demonstrated a replication rate of 86%, contrasting with the 50% replication rate typically reported. The improvement was attributed to “the use of best practices” and ensuring “all of the right steps are taken”. Ironically, the study described (Protzko et al. 2024a) was subsequently retracted after concerns were raised, in particular with respect to the preregistration of the meta‐study (Protzko et al. 2024b). Having access to the right tools is a start, and we recommend much wider uptake of the techniques described here, as well as independently by Beaulieu‐Jones and Greene (2017). However, the tools must be used correctly, and whatever benefits they offer to the scientific publication process, the need for the established processes of peer review, critique, corrections, and even retractions will remain. Nevertheless, the possibility of integrating reproducible analyses and continuous integration frameworks more closely with the peer review and publication processes is intriguing.

### 4.1 Aiding the Reviewer

Mistakes in scientific research are inevitable (hopefully arising through unintentional human errors rather than through intentional fraud); however, requiring that analyses are fully encoded and demonstrably reproducible at least guarantees that mistakes are discoverable (albeit with effort).

A fully encoded analysis that is demonstrably reproducible does not mean that the underlying code would be easy to review. Just as written text can be clear or unintelligible, so can computer code be obfuscated (Montfort 2008), either unintentionally or in a deliberate attempt to hide its purpose or methodology. A general adherence to coding standards and good practices may be required if code review is to be effective. An advantage of reviewing code within the kahawai.io system is that it can be tested with relatively little effort.

### 4.2 Examples of Bad Practices

The reproducibility of an analysis can be undermined if the Docker image is incomplete and dynamically loads additional software at the time of execution. In this case, the resulting software version may change over time. This practice can be prevented if the image is run in an environment that prevents access to the wider internet, or it can simply be discouraged. Making the effort to ensure all the required software is within the Docker image is a prerequisite for a fully reproducible analysis.

The perils of using spreadsheets for managing data are, we hope, well known, see, for example, (Sullivan 2019; Lewis 2021). Issues include the ability for data to be altered without preserving the original, and a lack of adherence to data types and layouts. Nevertheless, similarly bad practices are possible in fully encoded analysis, including assigning or changing values without justification, or making a poor decision around when an approach to fixing erroneous data points should be generalised, versus dealing with specific instances.

### 4.3 Other Aspects of Reproducibility

In the terminology proposed by Gundersen (2021), the reproducibility provided by the kahawai.io system, and related approaches, would probably be classified as *OR4*, indicating that the outcomes were the same when based on the text, code, and data provided in the original investigation. The kahawai.io system somewhat removes the distinction between the original and independent researchers because any user with access to the report can rerun the analysis.

We note that further evidence of reproducibility, in its wider senses, is provided by recoding of an analysis, applying alternative analysis techniques, and (of course) by collecting new or additional data.

Different researchers will not always interpret results in the same way; thus, the implementation of fully encoded analyses does not guarantee that results are “interpretation reproducible”.

Some of these different facets of reproducibility are illustrated by the process of updating standardised catch per unit effort (CPUE) series; these are frequently used as indices of abundance for fish stocks. These series involve statistical standardisation (i.e., removing effects of operational variables on the data to recover signals of relative abundance), in this case using generalised linear models of catch and fishing effort data reported to Fisheries New Zealand.

**FIGURE 3:**
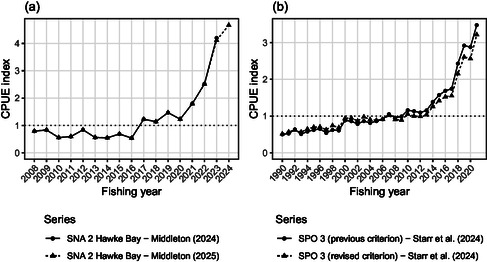
Updates of catch per unit indices for (a) snapper off the central east coast of the North Island (*Pagrus auratus*; SNA 2) and (b) rig off the east coast of the South Island (*Mustelus lenticulatus*; SPO 3).

For snapper off the central east coast of the North Island, abundance is monitored using tow‐by‐tow data from Hawke Bay (Middleton 2024), while rig (a shark species) off the east coast of the South Island is monitored using trip‐by‐trip data (Starr et al. 2024). The snapper analysis was originally implemented using the ghoti library on the kahawai.io platform, and was subsequently updated with a further year of data (Middleton 2025). This was achieved with a minor change to the analysis code (adjusting the end date to accommodate the new data), resulting in an updated analysis that was very close to the previous analysis, for the years in common (Figure 3a).

The rig analysis was also implemented on the kahawai.io platform, but updated a previous analysis that had been carried out independently. Working from the documented description of the previous analysis, we discovered that one aspect was described incorrectly; this related to the criterion used to select data for the analysis. In this case, the differences were minor (Figure 3b) with no consequences for the assessment of stock status (i.e., interpretation reproducible). An investigation of the different approaches, however, suggested the previously documented criterion had the potential to cause positive bias when stock abundance was low.

While the ease of updating the snapper series demonstrated the efficiency of reusing the same code, and the rig example illustrated the challenge of reimplementing analyses based solely on a description of the methods, we nevertheless acknowledge that recoding analyses to test their reproducibility can often be worthwhile, leading to new insights and improved methods.

### 4.4 Testing for Equivalent Outcomes

In our brief description of continuous integration approaches, we did not discuss the role of formal testing. In software development, some testing can be automated by describing the expected result of a particular piece of code and automatically checking that new code produces the same result. Building a suite of formal tests may have merit when applying a continuous integration approach to data analysis: for example, checks can be made on the input data set and on the numerical results of an analysis.

Pinto et al. (2018) describe potential issues with formal testing when applying continuous integration to software development. These include faulty tests and incomplete test suites, together with the potential for overreliance on passing tests that do not adequately cover the range of inputs or outputs encountered in practice.

In the case of statistical analyses, more thought needs to be given to developing tests for the numerical equivalence of results, especially in cases where sampling is employed (e.g., bootstrapping or MCMC sampling) and some variation in the results of repeatedly running the analysis is expected.

### 4.5 Repeatable Data Collection

In this paper, we have focused on the implementation of fully reproducible data analyses in situations where the source data have already been collected, often independently of the researchers undertaking the analysis. Nevertheless, we are sometimes involved in data collection programmes and consider that many of the disciplines involved in reproducible analyses can be extended to data collection programmes. The key lesson is that raw data should be preserved in their original form. This has two components:

(1) where data are transcribed from some other medium (e.g., paper forms, audio records, images), then these source records should also be preserved and, to the greatest extent possible, made available for inspection to resolve queries about the data; and
(2) to the greatest extent possible, changes (i.e., error corrections) to the data should be made via code that documents how the error was identified and why it was modified in a particular way. Where data are double‐entered, the originals should be retained and the reconciliation process recorded. Where it is absolutely necessary to make a change at the time of data entry (e.g., because the original is invalid), then the change should be noted in comments. Ideally, versioned copies of the data should be made where changes are made in the database, rather than through an encoded analysis step.

### 4.6 Open Source Code, Data Restrictions, and Open Access Publication

The kahawai.io system is implemented almost exclusively with open source software, and we rely on a variety of open source software when undertaking fisheries analyses. However, use of open source software is not a prerequisite for employing the reproducible analysis techniques we have described here; so long as the software can be incorporated in a Docker image, our approach can be implemented.

Many of the data inputs used in fisheries science are restricted or confidential because, for example, they include personal or commercially sensitive information. Similar restrictions exist in many other fields, such as medical research (Beaulieu‐Jones and Greene 2017). Fisheries New Zealand has established processes to permit researchers to use these data; as noted above, ensuring that our use of these data can be audited was one of the original motivations for developing the kahawai.io system. For independent researchers to reproduce any analyses of these data, whether using the original analysis code or a reimplementation, they will also have to have authorised access to the input data. Similarly, it is possible that not all the code used in an analysis will be able to be released publicly.

We do not see reasonable restrictions on data or code availability as fundamental barriers to the adoption of fully reproducible analyses as normal practice. Where reviewers need to scrutinise the code or data, then we would anticipate that researchers would facilitate this (voluntarily, or as a contract condition) and, ideally, collaborate in the process. Beaulieu‐Jones and Greene (2017) also discuss opportunities for the readers of the scientific literature to be provided with confidence in the results of reproducible analyses in situations where the source data are not public.

We consider that the implementation of fully reproducible analyses using platforms like the kahawai.io system is very relevant to discussions of open research and open access publishing (Saunders 2022). While these are not co‐dependent initiatives, it is likely that progress in both these areas will be required to re‐establish trust in the scientific publication process, including amongst practitioners, such as Bolland et al. (2025), who consider that there is little interest in trying to improve the situation.

### 4.7 Pathways to Reproducibility

Acknowledging that the implementation of fully reproducible analyses is not routinely taught to new researchers or required of current practitioners, we recognise that encouraging its uptake without appropriate incentives from publishers and institutions may be considered optimistic, despite the benefits we have documented. To conclude on a positive note, therefore, we emphasise that the implementation of fully reproducible analyses does not have to be adopted on an “all or nothing” basis, but can gradually be incorporated in one's work.

By way of example, we routinely use templates that allow us to reproducibly produce reports for Fisheries New Zealand in a “publication ready” format. Adopting these templates can be challenging for those who are more comfortable with the writing environment provided by modern word processors. Even for those experienced with use of the templates, the discipline of writing reports that reproduce calculations, rather than transcribe results from elsewhere, requires an additional investment of time. However, many of the benefits of reproducible analyses can be obtained by stepwise adoption: focussing first on the key analyses, and later on the full work‐flow. In time, the process of writing calculations, rather than results, in reports becomes more natural, often precipitated by recognising that failing to update key results in response to new data or new analyses can be avoided by writing code rather than transcribing numbers.

## Supporting Information

Additional supporting information can be found online in the Supporting Information section.

## Funding

No specific funding was received in support of the writing of this paper; it was undertaken by the authors in their roles as members of the Kahawai Collective. Members of Kahawai Collective fund its operations by financial contributions in proportion to use of the Collectives’ resources, and make in‐kind contributions. We acknowledge the original funding by members of the Trident Systems Limited Partnership, and Seafood Innovations Limited, that led to the initial development of the kahawai.io platform.

## Disclosure

All four authors are active participants in fisheries research in New Zealand and overseas, undertaking contracted projects on behalf of government (Fisheries New Zealand and the Department of Conservation), regional fisheries management organisations, seafood industry representative bodies, and individual seafood companies. All authors were involved in developing the concepts reported here. DM wrote the majority of the text, with assistance of F.T., A.L. and P.N. reviewed the text and assisted in refining the concepts.

## Supporting information

Supplementary Material

## Endnotes

1
We acknowledge that spreadsheets can often be automated by a macro language, but this is not their normal mode of operation. Such macro languages tend to be less capable than software that is designed to be driven by code, and are often poorly documented.

2
https://github.com.
