# Supplementary material for: Implementing Reproducible Fisheries Research: A Decade of Experience With the Kahawai Reporting System
Source: J R Soc N Z. 2026 Feb 4;56(1):e70011. doi: 10.1002/snz2.70011 (PMC12964991; doi:10.1002/snz2.70011)
Supplement: Supplementary file 1 — Supplementary Material [file SNZ2-56-e70011-s001.pdf]

SUPPLEMENTAL INFORMATION

Implementing reproducible fisheries research: a decade of experience with the kahawai reporting system. Supplemental Information.

David A. J. Middleton[a], Finlay N. Thompson[b], Adam D. Langley[c], Philipp Neubauer[b]

[a]Pisces Research Limited, Wellington, NZ; [b]Dragonfly Data Science, Wellington, NZ; [c]Trophia Limited, Nelson, NZ

ARTICLE HISTORY
Compiled March 21, 2025

1. Components of the kahawai.io system

1.1. *Database server*

The database server, while not a prerequisite for undertaking reproducible analyses generally, is a key component of the `kahawai.io` system. In the `kahawai.io` system, the database server uses the PostgreSQL open source, object-relational database system that uses and extends the SQL language. In particular, the PostGIS extensions add support for storing, indexing, and querying geospatial data. The `kahawai.io` database server allows controlled access to a number of fisheries datasets, many of which are built from extracts provided by Fisheries New Zealand from the range of fisheries research databases they administer.

1.2. *Control interface*

The running of analyses (usually referred to as ‘reports’ in the `kahawai.io` system) is managed from the control interface, which includes a web-based user interface. This system interacts with the GitHub code repository for an analysis, and the job runner. Access to the control interface is restricted to authorised users, and the ability to run and inspect the outputs of a specific analyses is managed through access controls on the specific GitHub repository that hosts the analysis code.

1.3. *Job runner*

The job runner implements the continuous integration approach described above. It looks for reports to be re-run, which may be triggered manually or automatically, such as when the code changes. It then manages the setup and completion of report jobs. A key part of this process is running the analysis code with the required `Docker` image but, in the `kahawai.io` case, it also allows each job to be run in a carefully defined

CONTACT D. A. J. Middleton. Email: `david@pisces.nz`

CONTACT D. A. J. Middleton. Email: `david@pisces.nz`, F. N. Thompson. Email: `finlay@dragonfly.co.nz`, A. D. Langley. Email: `adam_langley@xtra.co.nz`, P. Neubauer. Email: `philipp@dragonfly.co.nz`

context with managed access to databases and networks. The `kahawai.io` system is agnostic with respect to the programming language or software tools that are used for an analysis; the only requirement is that the required tools are made available through a `Docker` image.

2. Using the kahawai.io system

2.1. *Conveniences that build consistency*

The development of efficient and collaborative approaches to the implementation of reproducible analyses often results in the establishment of particular patterns, in terms of the tools used, and the way code is laid out. For example, while it is unnecessary for the single command that launches an analysis to have a fixed name, we have found that adopting consistent naming (`build.sh`, in our infrastructure) supports the process of producing transparently reproducible analyses by removing one, unnecessary, source of variation.

3. Maintaining the kahawai.io system

3.1. *The evolution of the Kahawai Collective*

The development of the `kahawai.io` system was originally funded by the Trident Systems Limited Partnership ('Trident'), an organisation established by a group of fisheries quota owners to develop and apply innovative systems and processes that supported effective management of New Zealand fisheries. One of Trident's principal work streams focussed on getting greater value from fisheries data, and Dragonfly Data Science was contracted to build a reporting system. This was originally intended to provide the Ministry for Primary Industries with the ability to audit the use of statutory catch and effort data that were supplied to assist in Trident's implementation of catch sampling programmes. The implementation drew on Dragonfly's experience in applying modern software practices to data analysis problems.

The resulting reporting system was found to have value beyond the support of catch sampling programmes. The ability to efficiently and repeatedly apply standardised data preparation procedures to new datasets encouraged work to implement a range of approaches that were commonly used to prepare New Zealand fisheries data for analysis (Bentley 2012). In parallel, the contributors to these developments found they supported further efficiencies in their own work, and the concept of the system as a "club good" evolved, with the system co-developed and supported by its users.

Following the disestablishment of Trident (see ICES 2023, box 1, for the background on this decision), responsibility for the `kahawai.io` system passed to Pisces Research Limited. Recognising the co-development involved in the establishment of the system, and the efficiencies and other benefits implicit in the informal "club goods" approach, we opted to establish the Kahawai Collective as custodian of the `kahawai.io` system. The Kahawai Collective is a non-profit group of fisheries research providers that work together to develop and maintain tools and resources, and act as data custodians for a range of datasets, that support efficient research into New Zealand fisheries and marine ecosystems.

4. Wider adoption of the framework

The control interface used by the `kahawai.io` system was originally codenamed `gorbachev` because of the focus of providing a trusted, and verifiable, framework for data use. It has subsequently been renamed `Gateaux`, reflecting the importance of 'recipes' (computer code) in producing reproducible data analyses, and the benefits in doing this in an open and transparent manner (and encapsulated in the 'made to share' motto of `Gateaux`). As noted in the main text, the `Gateaux` infrastructure has been adopted by a number of other entities.

Many of the tools that are used in the `kahawai.io` system were unfamiliar to both fisheries scientists and data scientists when the system was developed. Today, their use is becoming more common. For example, the GitHub version control system is also widely used by fisheries organisations, including the International Council for the Exploration of the Sea, the US National Oceanic and Atmospheric Administration, the North Pacific Fisheries Commission, and the secretariat of the Southern Indian Ocean Fisheries Agreement.

References

Bentley, N. 2012. "Groomer: grooming and other things for New Zealand fishstocks." `https://github.com/trophia/groomer`.

ICES. 2023. "Workshop on developing guidance for ensuring the integrity of scientific information submitted to ICES by data providers (WKEnsure)." *ICES Scientific Reports* 5:44. https://doi.org/10.17895/ices.pub.22692058.
